# Development of a circulation direct sampling and monitoring system for O_2_ and CO_2_ concentrations in the gas–liquid phases of shake-flask systems during microbial cell culture

**DOI:** 10.1186/s13568-017-0464-4

**Published:** 2017-08-23

**Authors:** Masato Takahashi, Yoshisuke Sawada, Hideki Aoyagi

**Affiliations:** 1 Division of Life Sciences and Bioengineering, Graduate School of Life and Environmental Sciences, University of Tsukuba, Tsukuba, Ibaraki 305-8572 Japan; 2 Iwashiya Bio Science, Inc., 2-18-4, Higashi Shinmachi, Itabashi-ku, Tokyo, 174-0074 Japan; 3 Faculty of Life and Environmental Sciences, University of Tsukuba, Tsukuba, Ibaraki 305-8572 Japan

**Keywords:** CO_2_, *Escherichia coli*, Headspace, Real-time monitoring, Sampling, Shake-flask culture

## Abstract

Monitoring the environmental factors during shake-flask culture of microorganisms can help to optimise the initial steps of bioprocess development. Herein, we developed a circulation direct monitoring and sampling system (CDMSS) that can monitor the behaviour of CO_2_ and O_2_ in the gas–liquid phases and obtain a sample without interrupting the shaking of the culture in Erlenmeyer flasks capped with breathable culture plugs. Shake-flask culturing of *Escherichia coli* using this set-up indicated that a high concentration of CO_2_ accumulated not only in the headspace (maximum ~100 mg/L) but also in the culture broth (maximum ~85 mg/L) during the logarithmic phase (4.5–9.0 h). By packing a CO_2_ absorbent in the gas circulation unit of CDMSS, a specialised shake-flask culture was developed to remove CO_2_ from the headspace. It was posited that removing CO_2_ from the headspace would suppress increases in the dissolved CO_2_ concentration in the culture broth (maximum ~15 mg/L). Furthermore, the logarithmic growth phase (4.5–12.0 h) was extended, the U.O.D._580_ and pH value increased, and acetic acid concentration was reduced, compared with the control. To our knowledge, this is the first report of a method aimed at improving the growth of *E. coli* cells without changing the composition of the medium, temperature, and shaking conditions.

## Introduction

Shake-flask cultivation in Erlenmeyer flasks with breathable culture plugs was first developed in 1932 for the submerged culture of *Aspergillus niger* (Kluyver and Perquin 1933). Shake-flask culture is frequently used to screen for secondary metabolites and optimise culture conditions for microorganisms in the initial steps of bioprocess development, because a lot of samples can be aerobically batch cultured in parallel at a low cost (Van Gool et al. 2011; Diederichs et al. 2014). In evaluating cultured samples, it is important to understand the culture environment (e.g., dissolved O_2_ and CO_2_, pH, biomass, nutrition source, products, oxidation–reduction potential, etc.) during the shake-flask culture of microorganisms. However, since Erlenmeyer flasks (with culture plugs) containing culture broth are rotated at a high speed, monitoring multiple culture factors in both the headspace and culture broth simultaneously is difficult.

The monitoring of environmental factors in the culture broth during shake-flask culture has been reported previously. A fluorescent dye sensor affixed to the inner wall of the flask, in combination with an irradiator and detector, can be used to measure the pH (Scheidle et al. 2007; Schneider et al. 2010) and dissolved O_2_ (Schneider et al. 2010; Flitsch et al. 2016). However, incorporating multiple sensors, irradiators, and detectors in the flask and shaking table platform is unrealistic owing to the small size of the flask.

The CITSens Bio-sensor has been developed to monitor glucose and lactic acid in the culture broth. The device uses enzymatic sensors integrated into a non-breathable cap instead of breathable culture plugs (Bauer et al. 2012). However, this method does not allow the monitoring of environmental factors in the culture broth during shake-flask culture using Erlenmeyer flasks with culture plugs.

Currently, to monitor the environmental variables, several sample flasks are prepared and cultured simultaneously, and a single flask is retrieved from the shaking table platform periodically and sampled aseptically to measure the time course of the shake-flask culture of microbes. However, in this method, several flasks need to be prepared under similar conditions. Moreover, the sampled culture broths originate from different flasks. Therefore, the results obtained by this method are not measured over the same time course as in the case of an Erlenmeyer flask with a culture plug. Thus, deviations of the measured data tend to occur.

To our knowledge, there are no reports on the effect of temporary interruption of shaking by the sampling operation on the kinetics of microbial cell culture. Generally, during shake-flask culture, O_2_ supply is limited, and the dissolved O_2_ concentration in the broth tends to be depleted during microbial cell cultivation (Henzler and Schedel 1991). Temporary interruption of shaking will stop the supply of O_2_ into the culture broth, making it difficult to maintain aerobic culture conditions, which can affect cell respiration. Therefore, in order to minimise the influence of manual operations such as sampling on shake-flask culture and to understand the effects of various culture environments, it is important to procure samples from the same Erlenmeyer flask without interrupting the shaking.

Monitoring of culture conditions in the headspace of shake-flasks using the OTR-Device (Anderlei and Büchs 2001) and RAMOS [Respiration Activity Monitoring System] (Anderlei et al. 2004) has been reported previously. These devices measure the oxygen transfer rate and respiration activity, respectively. However, these systems do not directly monitor CO_2_ and O_2_ in the headspace of Erlenmeyer flasks with breathable culture plugs during microbial cell culture. The BCpreFerm system for shake-flasks (BlueSens, Herten, NW FRG) is an online monitoring device for shaken culture systems, which can be equipped with standard culture plugs. However, this device can monitor only in the gas phase of shake-flasks, and the sampling operation involves temporary interruption of shaking.

Therefore, we aimed to develop the following two systems: (A) a direct monitoring system of culture conditions in shake-flask culture using culture plug-capped Erlenmeyer flasks, and (B) a system that can continue sampling from the same flask without interruption of shaking by using system (A). By using system (A) or (B) appropriately, one can measure various culture factors directly in both the culture broth and headspace of the shake-flask during microbial cell cultivation.

We attached a gas–liquid two-phase circulation system to an Erlenmeyer flask with culture plug. The new system (termed circulation direct monitoring and sampling system [CDMSS]) can directly measure CO_2_ and O_2_ concentrations in both the culture broth and the headspace. CDMSS can sample culture broth without interrupting the shake-flask culture. With this device, CO_2_ and O_2_ concentrations were monitored and various culture factors (pH, U.O.D., and acetic acid concentration) were measured in the shake-flask cultivation of *Escherichia coli*. We also used CDMSS to analyse the influence of CO_2_ produced by *E. coli* cells on their growth during shake-flask culture. Thus, we have developed a monitoring system that allows the improvement of culture conditions by focussing on CO_2_ produced in shake-flask culture using culture plug-capped Erlenmeyer flasks, and provides useful culture data for the initial steps of bioprocess development.

## Materials and methods

### Circulation direct monitoring and sampling system for shake-flask (CDMSS)

**Fig. 1:**
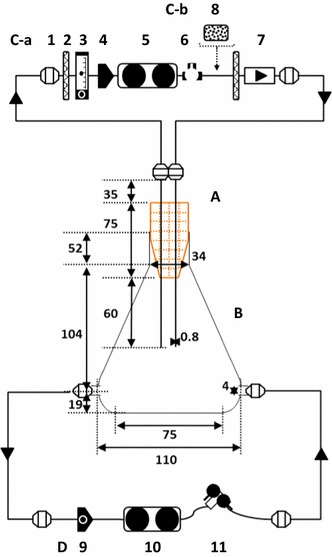
Schematic flow diagram of the circulation direct monitoring and sampling system for shake-flask (CDMSS). *A* Culture plug penetrated with a needle; *B* Erlenmeyer flask with branches on the bottom; *C-a* the gas circulation system (normal); *C-b* the gas circulation system (with CO_2_ absorbent); *D* the liquid circulation system; *1* connector; *2* 0.22 µm filter; *3* flow meter; *4* diaphragm-type pump; *5* liquid measurement unit; *6* gas sampling unit; *7* check valve; *8* column packed with CO_2_ absorbent; *9* peristaltic pump; *10* liquid measuring unit; *11* liquid sampling unit

The monitoring system developed in this study, CDMSS, allows the analysis of various culture factors in the gas–liquid phases during shake-flask culture using a breathable culture plug-capped Erlenmeyer flask without interrupting the shaking (Fig. 1). We used an Erlenmeyer flask that was branched on the bottom, and a needle (21G) penetrated the culture plug. The CDMSS used an existing shaker and shaking table platform, and a gas–liquid two-phase circulation system, including the monitoring and sampling units. Circulation was maintained at a constant flow rate by using a tubing in the headspace and a pump in the culture broth in the flask (with branches on the bottom) to monitor the behaviour of CO_2_ and O_2_.

Hydrophobic filters with a pore size of 0.22 μm (Advantec Co., Ltd., Ehime, Japan) and a check valve were added to the gas-phase circulation system to prevent contamination (Fig. 1). In addition, a CO_2_ absorbent, YABASHI LIME (Yabashi Industries Co., Ltd., Gifu, Japan), was packed in the headspace of the flask to remove CO_2_ (Fig. 1).

The measuring unit consisted of a special holder and equipment for measuring the CO_2_ and O_2_ concentrations. The CO_2_ and O_2_ concentrations in the headspace of the culture flask were measured in real time using a GMT221 sensor (Vaisala Oyj, Helsinki, Finland) and a FOM-2000 sensor (ASR, Tokyo, Japan), respectively. Dissolved CO_2_ and O_2_ concentrations in the culture broth were measured in real time using InPro5000i and InPro6850i sensors (Mettler Toledo, Columbus, OH, US), respectively. In measuring the dissolved CO_2_ concentration using InPro5000i, there was no cross interference of volatile organic acids produced simultaneously by the cells during fermentation. Calibration of all equipment, including measuring instruments in the CDMSS, was carried out according to the manufacturers’ instructions. The values measured by CDMSS were visually monitored using a video camera over time because the CDMSS was not equipped with a data logger.

A stainless-steel two-way cock and disposable three-way stoppers were used for the sampling units of the liquid and gas phases, respectively. Sampling of the culture broth was performed using a disposable syringe from the sampling port of the two-way cock after sterilizing with 70% ethanol and flame. After sampling, the two-way cock was covered again with aluminium foil and sterilised (70% ethanol and flame) to prevent contamination in the circulation system.

All shake-flask cultures involving the CDMSS were performed at a liquid-phase circulation rate of 15 mL/min and a gas-phase circulation rate of 400 mL/min. The time that air and culture medium spent circulating in CDMSS was very short (about 8 s). No sedimentation or clogging of the cells was observed in the circulation system of the liquid phase during shake-flask culture.

### Microorganisms and medium

*Escherichia coli* K12, strain IFO3301, was used in this study. The LB medium used to culture *E. coli* consisted of (g/L): tryptone, 10; yeast extract, 5; and NaCl, 5.

### Inoculum preparation

A loop-full of *E. coli* strain IFO3301 slant culture was inoculated into a 500 mL Erlenmeyer flask containing 100 mL of LB. The sample was then cultured at 30 °C on a rotary shaker at 200 rpm and 70 mm shaking diameter for 6.75 h. Glycerol stocks were prepared by adding the culture medium to glycerol (final concentration 15% [v/v]) and stored at −80 °C.

### Culture conditions

In this study, we used Erlenmeyer flasks for culture. One millilitre of each glycerol stock was inoculated into a 500 mL Erlenmeyer flask containing 100 mL of LB and cultured at 30 °C on a rotary shaker at 200 rpm and 70 mm shaking diameter. For air permeability, the Erlenmeyer flask was equipped with a BIO-SILICO N-38 sponge plug (Shin-Etsu Polymer Co., Ltd, Tokyo, Japan; breathable culture plug).

### Measurement of culture factors

**Fig. 2:**
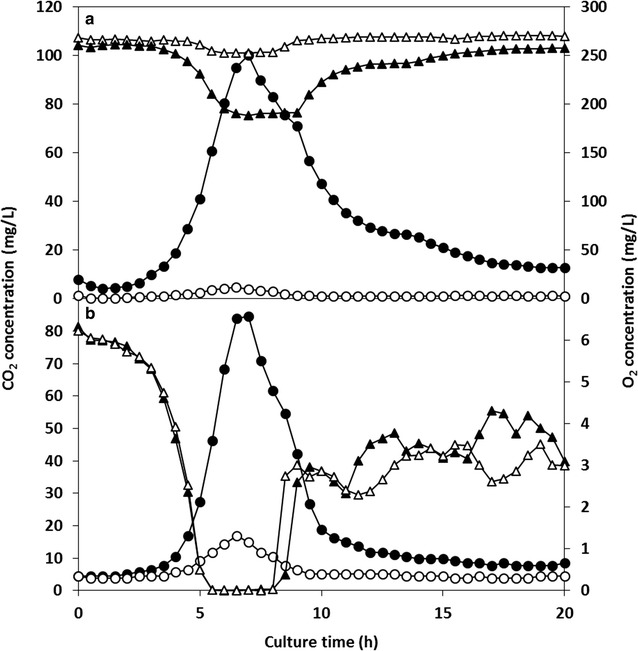
Real-time CO_2_ and O_2_ concentrations in normal and specialised shake-flask culture of *E. coli* IFO3301. **a** In the headspace; **b** in the culture medium. Symbols: *circles* oxygen; *triangles* carbon dioxide; *closed* normal shake-flask culture; *open* specialised shake-flask culture (with removal of CO_2_ in the headspace). Shake-flask culture conditions were 100 mL of LB medium at 30 °C, 200 rpm shaking frequency and 70 mm shaking diameter, 500 mL Erlenmeyer flask capped with a culture plug and equipped with CDMSS

**Fig. 3:**
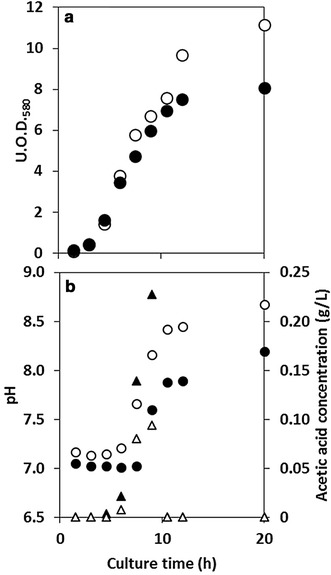
Changes in culture factors in the two shake-flask cultures. **a** Cell biomass (U.O.D._580_); **b** pH and acetic acid concentration. Symbols: *closed circles* control (normal shake-flask culture); *open circles* CO_2_ absorbent-equipped (specialised shake-flask culture). Symbols in **b** *circles* pH; *triangles* acetic acid concentration

The concentrations of CO_2_ and O_2_ in the gas–liquid phases during shaking culture were monitored using CDMSS. The U.O.D._580_, pH, and acetic acid concentration were measured from the culture broth, which was sampled without interruption of shaking, using a V-570 spectrophotometer (JASCO, Tokyo, Japan), pH meter (HORIBA, Kyoto, Japan), and acetic acid f-kit (R-Biopharm AG, Darmstadt, LH FRG), respectively. In order to ensure minimal decrease in the volume of the culture broth owing to sampling from the same flask, the total sampling volume was maintained at <10% of the total amount of the initial culture medium. All experiments were performed in duplicate. The results are expressed as means in Figs. 1, 2 and 3, they were confirmed to be highly reproducible (only the value of dissolved oxygen concentration fluctuated slightly at the latter stage of culture [after about 10 h of cultivation] in Fig. 2b).

## Results

The results of the monitoring of O_2_ and CO_2_ concentration in the gas–liquid phases of the flask and the measurement of U.O.D._580_, pH, and acetic acid concentration are shown in Figs. 2 and 3, respectively.

The dissolved O_2_ concentration decreased gradually until 3 h, then declined sharply, and was depleted between approximately 5 and 8 h (Fig. 2b). Then, the dissolved O_2_ concentration suddenly began to increase to approximately 3 mg/L and was maintained at approximately 3–4 mg/L. The O_2_ consumption rate of *E. coli* might have decreased because of nutrient depletion from the LB medium.

The CO_2_ concentration did not change significantly until 3 h, then rose sharply, reached a maximum of 85 mg/L, and dropped dramatically thereafter (Fig. 2b). This may be explained as follows: *E. coli* catabolised the initial nutrient supply, proliferated rapidly, and then remained in the stationary phase after nutrient depletion.

The behaviours of O_2_ and CO_2_ in the headspace were similar to those in the culture broth (Fig. 2a). The O_2_ in the headspace was not depleted but decreased to 190 mg/L, and the CO_2_ concentration in the headspace rose to over 100 mg/L. The U.O.D._580_ of *E. coli* rose exponentially between approximately 4.5 and 9.0 h, and the pH increased between 6 and 9 h.

The following observations were noted in the control (shaking culture of *E. coli* using an Erlenmeyer flask): (a) dissolved O_2_ was almost depleted in the logarithmic growth phase (approximately 4.5–9.0 h), and (b) *E. coli* cells were exposed to high concentrations of dissolved CO_2_ (the maximum value was 84.5 mg/L) compared with the initial culture condition (4.0 mg/L). Some of the CO_2_ produced by respiration of *E. coli* cells could not be dissolved in the culture broth, and was consequently discharged into the headspace of the flask, where it accumulated on account of insufficient ventilation through the culture plug. The partial pressure and amount of dissolved CO_2_ increased according to Henry’s law (Fig. 2).

High concentrations of CO_2_ accumulated in both the headspace and the culture broth in actively proliferating microbes during normal shake-flask culture. Dissolved CO_2_ has been shown to suppress cell growth in aerobic culture (Dixon and Kell 1989). Therefore, we hypothesised that the accumulating CO_2_ in the headspace of the shake-flask culture suppressed the growth of *E. coli* cells, and the removal of CO_2_ from the headspace might offset the increase in dissolved CO_2_. In order to remove the accumulated CO_2_ from the headspace, the circulation system of the headspace was changed from C-a to C-b in Fig. 1.

In the case of C-b in Fig. 1, the CO_2_ in the headspace could be maintained at a very low concentration throughout the cultivation period; the maximum value was < 5.0 mg/L (Fig. 2a). The O_2_ concentration in the headspace was maintained at a higher level (~250 mg/L) compared with the control (normal shake-flask culture). However, the behaviour of the O_2_ concentration in the headspace was similar to that of the control. The dissolved O_2_ concentration was similar to that of the control (Fig. 2). The dissolved CO_2_ in the culture broth could be maintained at a very low concentration; the maximum value (~17 mg/L) was one-fifth that of the control (Fig. 2b).

Thus, removing CO_2_ from the headspace of the flask suppressed the increase in dissolved CO_2_ concentration in the culture broth. Furthermore, the logarithmic growth phase (4.5–12.0 h) was extended and the U.O.D._580_ value increased compared with the control (Fig. 3a). Of note, the pH of the culture broth also increased compared with the control (Fig. 3b). This may be because the pH buffering capacity decreased owing to the decrease in dissolved CO_2_. The concentration of acetic acid was higher in the normal shake-flask culture than in the specialised culture (Fig. 3b).

## Discussion

The monitoring system developed in this study has the following two advantages: (1) it allows the direct monitoring of CO_2_ and O_2_ in the gas–liquid phases during shake-flask culture using culture plug-capped Erlenmeyer flasks, and (2) it allows sampling without interruption of shaking. To our knowledge, there are no reports of real-time monitoring of CO_2_ and O_2_ in both the gas phase and the liquid phase (culture broth) of breathable plug-capped Erlenmeyer flasks during standard shake-flask culture. Further, sampling without interruption of shaking has not been reported in conjunction with the conventional shake-flask cultivation method. By using a filter (pore size 0.22 µm), the gas phase of CDMSS was circulated through a packed CO_2_ absorbent, and CO_2_ generated in the headspace of the flask during shaking culture was removed.

Generally, under high-speed shaking conditions, such as those of shake-flask culture, there is a tendency for the measured values in the gas–liquid phases to drift if the measurement equipment is directly connected to the flask during measurements (Vasala et al. 2006; Ruottinen et al. 2008). In this regard, CDMSS offers an advantage in terms of measurement accuracy because it does not shake the measurement equipment and hence maintains gas–liquid contact with the measurement site, and the gas and liquid remain at a constant speed. It is important to note that the measurement site should be in contact with the culture medium while keeping the baffle effect in mind: the pipe provided at the bottom of the flask to circulate the culture medium has a baffle effect (increase in *k*_L_ *a*) if its diameter is too large. If the cultured microbes form pellets, it is necessary to prevent them from clogging the CDMSS.

The CDMSS provides various forms of information about the culture environment in the culture broth and headspace during shake-flask culture. The depletion of dissolved O_2_ in the logarithmic growth phase has been reported previously (Vasala et al. 2006; Schmidt-Hager et al. 2014). In our study, the dissolved oxygen concentration value fluctuated after about 10 h, and the cause of this is unknown. To our knowledge, this is the first study to report that the CO_2_ concentration increases (~85.0 mg/L) during shake-flask culture using a culture plug-capped Erlenmeyer flask, compared with the initial culture condition (~4.0 mg/L). A previous study has reported the monitoring of dissolved CO_2_ in shake-flask culture (Ge and Rao 2012). However, this study focused on the development of a sensor for dissolved CO_2_ and O_2_, and the pH in the flask was different from that at standard conditions of shake-flask culture (a milk filter and 2 L flask filled with 50 mL of medium).

There are a number of reports on the influence of CO_2_ on microbial cell growth in shake-flask culture (Repaske et al. 1971; Repaske and Clayton 1978; Kato and Tanaka 1998). The cell yield of *Saccharomyces cerevisiae* was 20% lower in Erlenmeyer flasks than in aerated flasks (fresh air was aerated at 2.0 vvm) (Kato and Tanaka 1998). Repaske and Clayton (1978) reported that the growth of *E. coli* cells in shake-flask culture is dependent on the aeration of CO_2_ at low concentrations (0.005–0.3% v/v). *E. coli* requires a small amount of CO_2_ to grow (Brown and Howitt 1969). The CO_2_ in the central metabolic pathway of *E. coli* (a facultative anaerobic microbe) cannot be replaced (Merlin et al. 2003). The aeration of shake-flasks could have various influences other than removing CO_2_ (e.g., O_2_ supply and/or volatilisation of medium). The types and concentrations of aerated gas influence the microbial cell culture. Figure 3 shows the effect of the removal of CO_2_ from the headspace of the flask on *E. coli* cell culture in isolation, without the influence of other effects caused by aeration.

This is the first report that the CO_2_ produced by the respiration of *E. coli* accumulates in Erlenmeyer flask cultures capped with breathable culture plugs. The accumulated CO_2_ was enough to inhibit the growth of *E. coli* during shake culture.

CO_2_ can stimulate or inhibit the growth of microorganisms (Dixon and Kell 1989), and as the concentration of dissolved CO_2_ increases, the growth of *E. coli* is inhibited. Further, acetate accumulates constantly when the concentration of dissolved CO_2_ is maintained between 20 and 300 mbar by manipulating the inlet gas composition during batch culture in bioreactors (Baez et al. 2009).

It has been suggested that CO_2_ produced by respiration affects both the physiological activity of microbes (e.g. proliferation, metabolism, etc.) and the pH of the culture broth in shake-flask culture. Phosphate-buffered LB medium limits the pH increase compared with normal LB medium, but this has practically no effect on the final cell concentration (Losen et al. 2004). This finding suggests that the limiting factor of cell growth in LB medium during shake-flask culture is not the buffering capacity of the medium, but the dissolved CO_2_ concentration, which depends on the CO_2_ concentration in the headspace of the flask.

The findings in this study were obtained from shake-flask culture using breathable plug-capped Erlenmeyer flasks. This set-up is widely used in the early stages of small-scale culture, but not in relatively large-scale bioreactors. The outcomes of this study have three potential applications: (1) the set-up described here allows the control of headspace environment in shake-flasks capped with culture plugs, (2) the device allows the characterisation of conditions associated with various types of flasks and culture plugs, and (3) our findings provide a basis for developing new flasks and plugs for the optimisation of culture conditions.

Our device might aid in the scale-up of small-scale culture to bioreactors, as well as in the screening of culture-dependence of microorganisms, identification of useful metabolites, and screening for new microbes.

The CDMSS allows the determination of the volumetric oxygen transfer coefficients of various types of flasks and of gas transfer resistance through various types of culture plugs. CDMSS can also be used to monitor various gases (e.g., hydrogen, ammonia, methane etc.) by changing the measuring units of the device. To our knowledge, this is the first study to monitor the CO_2_ and O_2_ concentrations in both the headspace and the culture broth of Erlenmeyer flasks capped with culture plugs during shake-flask culture.

## Abbreviation

CDMSS: circulation direct monitoring and sampling system
