# Erratum to: Development of a circulation direct sampling and monitoring system for O_2_ and CO_2_ concentrations in the gas–liquid phases of shake‑flask systems during microbial cell culture

**DOI:** 10.1186/s13568-017-0481-3

**Published:** 2017-09-18

**Authors:** Masato Takahashi, Yoshisuke Sawada, Hideki Aoyagi

**Affiliations:** 1 Division of Life Sciences and Bioengineering, Graduate School of Life and Environmental Sciences, University of Tsukuba, Tsukuba, Ibaraki 305‑8572 Japan; 2 Iwashiya Bio Science, Inc., 2‑18‑4, Higashi Shinmachi, Itabashi‑ku, Tokyo, 174‑0074 Japan; 3 Faculty of Life and Environmental Sciences, University of Tsukuba, Tsukuba, Ibaraki 305‑8572 Japan

## Abstract

Following publication of the original article (Takahashi et al. 2017), the authors reported that there was a mistake in the legend of Figure 2.

## Erratum to: AMB Expr (2017) 7:163 DOI 10.1186/s13568-017-0464-4

Following publication of the original article (Takahashi et al. 2017), the authors reported that there was a mistake in the legend of Figure 2, which in the published article reads as follows:

Figure 2 Real-time CO_2_ and O_2_ concentrations in normal and specialised shake-flask culture of *E. coli* IFO3301. **a** In the headspace; **b** in the culture medium. Symbols: *circles* oxygen; *triangles* carbon dioxide; *closed* normal shake-flask culture; *open* specialised shake-flask culture (with removal of……………..

However, the sentence should read:

Figure 2 Real-time CO_2_ and O_2_ concentrations in normal and specialised shake-flask culture of *E. coli* IFO3301. **a** In the headspace; **b** in the culture medium. Symbols: *triangles* oxygen; *circles* carbon dioxide; *closed* normal shake-flask culture; *open* specialised shake-flask culture (with removal of……………..
